# Associations between teacher–parent relationships and preschool children’s social behavior problems—the chain mediating roles of work–family conflict and parenting self-efficacy

**DOI:** 10.3389/fpsyg.2024.1349652

**Published:** 2024-07-12

**Authors:** Guolei Liu, Zhen Jin, Xinhong Zheng, Zixian Wang, Weina Liu

**Affiliations:** ^1^Hebei Institute of International Business and Economics, Qinhuangdao, Hebei, China; ^2^School of Physical Education & Health, Wenzhou University, Wenzhou, Zhejiang, China; ^3^Psychological Crisis Intervention Center, 984th Hospital of the People's Liberation Army, Beijing, China; ^4^Cangzhou Preschool Teachers College, CangZhou, Hebei, China

**Keywords:** preschool children, parent–teacher relationships, work–family conflict, parenting self-efficacy, social behavior problems

## Abstract

In the Chinese cultural context, the collaborative interaction characteristics among three key entities - families, kindergartens, and communities - and the mechanisms of their correlation with preschool children’s social behavior problems have not been fully understood yet. Based on ecological systems Theory and social support theory, this study aimed to examine the correlation between parent-teacher relationships and preschool children’s social behavior problems in Chinese kindergartens, as well as the mediating role of parents’ work–family conflict and parenting self-efficacy. Structural equation modeling was used to test the research hypotheses based on a questionnaire survey of 1,784 parents of preschool children. The main findings of this study are as follows: (1) Parents’ perceived positive parent-teacher relationships are negatively correlated with preschool children’s social problems. (2) Parents’ work–family conflict and parenting self-efficacy mediate the relationship between parent-teacher relationships and preschool children’s social behavior problems; (3) Parents’ work–family conflict and parenting self-efficacy play a chain mediating role in the influence of the parent-teacher relationship on preschool children’s social behavior problems. Taken together, the results collectively further elucidate the correlation between parent-teacher relationships and preschool children’s social behavior problems, while also discussing other relevant factors pertaining to children’s social behavior problems. Theoretically, this study expands the understanding of how external environmental resources interact with home and family education. Practically, this research indicates that governments, early childhood education institutions, and workplaces need to strengthen their support for family education of preschool children. The findings contribute to promoting a multi-faceted co-operation aimed at enhancing the quality of early childhood education and fostering the social adaptability and holistic development of preschool children.

## Introduction

In 2020, the Preschool^1^ Education Law of the People’s Republic of China (Consultation Paper) mandated the participation of multiple entities, including kindergartens, families, and communities, in preschool education. It explicitly states that “kindergartens should actively communicate with parents or other guardians about the physical and mental development of children and guide them in implementing scientific parenting methods.” Additionally, it emphasizes that “the whole society should create a favorable environment for age-appropriate children to receive preschool education and grow up healthily.” In October 2021, the Family Education^2^ Promotion Law of the People’s Republic of China set out clear requirements for “family responsibility,” “state support,” and “social coordination” in children’s education.

The social development of preschool children refers to the process by which children, in the course of participating in social life, form emotions, attitudes and values that meet the requirements of society, master social interaction skills, implement actions based on social norms, and gradually develop from a biological person into a social person (Pei and Li, 2020). Early childhood is a sensitive period for interpersonal relationships. It is an important part of the overall development of children, and the promotion of children’s social ability development is one of the core objectives of preschool education. Social skill development is critical to children’s psychological and physical development. It serves as the foundation for children’s social adaptation, likely enhancing the quality and effectiveness of children’s social interactions and adaptability, thereby resulting in a reduction of behavioral problems (Winsler and Wallace, 2002).

Family, kindergarten, and community are intertwined to form the ecosystem of preschool children, and all of them have an important influence on the social ability development of preschool children. Most research findings on the synergy between families, kindergartens, and the community in preschool education are still primarily in the realm of theoretical construction, with relevant empirical studies being relatively scarce. Specifically, there is a noticeable insufficiency in research focusing on the shared mechanisms through which various ecological environments, like family, kindergarten, and community, impact the social development of preschool children. Therefore, this study employed a quantitative research paradigm to investigate and analyze the relationship between teacher-parent relationships, parents’ work–family conflict, parenting self-efficacy, and preschool children’s social behavior problems. It aims to provide guidance for families, kindergartens, and communities to collaboratively foster children’s growth, while also offering empirical evidence to assist the state in supporting childbearing and constructing a child-friendly society.

### Teacher–parent relationships and preschool children’s social behavior problems

Through participation in social life, preschool children progressively acquire social interaction skills, develop initial aspirations, feelings, and attitudes that align with societal expectations, learn to adhere to social norms, gain experience in social roles, and establish foundational interpersonal relationships (Tang and Yao, 2017). The age range of 3– 6 years is crucial for an individual’s social development, characterized by immaturity and rapid changes, and it is a phase particularly susceptible to social adaptation challenges. Preschool children’s social behavior problems represent a form of social dysregulation, primarily characterized by immature responses in emotional and social adaptation (Achenbach et al., 1987). This includes both external behavior problems, such as aggression and provocative actions, and internal problems, like depression, anxiety, and social withdrawal (Achenbach, 1991). Numerous studies indicate that social behavior problems emerging during preschool years often persist and adversely affect individuals’ long-term development (Liang et al., 2022). For instance, preschool children’s social behavior problems are predictive of lower academic abilities or achievements in the future (Bulotsky-Shearer and Fantuzzo, 2011). Therefore, it is necessary to explore both the protective factors aiding preschool children’s social development and the risk factors contributing to social behavior problems. Clarifying the correlation between various factors and preschool children’s social behavior problems is important for promoting children’s social development. This will not only enhance their social competence but also protect their physical and mental well-being, ensuring their holistic development.

According to Bronfenbrenner’s ecological systems Theory (Bronfenbrenner et al., 1993), both the family and the kindergarten are the microsystems that influence the development of preschool children. First, children acquire social skills through direct contact with family members. Factors such as a family’s economic and social status, the dynamics among its members, and the quality of the parental marriage are closely related to preschool children’s social behavior problems (Dadds and Powell, 1991; Koot, 1993). Kindergarten is another microsystem that influences the preschool children’s social development. In this environment, the interactions between teachers and children, as well as among peers, play a pivotal role in shaping children’s social interactions. Furthermore, the interactions between the two microsystems of the family and the kindergarten form intermediate systems. Children’s social development may achieve a higher level when these microsystems are connected through strong, positive relationships (Liu and Meng, 2009). Therefore, the two microsystems, family and kindergarten, cannot be considered in isolation (Smith et al., 2000). The synergistic influence of the family and kindergarten play a crucial role in elevating the quality of preschool education and in the holistic physical and mental growth of children (Albright and Weissberg, 2010). In the process of interaction and co-operation between the family and the kindergarten, an individual forms a psychological perception of the emotional character and quality of the relationship, known as the “parent-teacher relationship” (Vickers and Minke, 1995). This study focuses on examining both the observable actions and personal perspectives in parent-teacher interactions. It emphasizes that strong parent-teacher relationships significantly contribute to children’s academic success and social skills (Hill and Taylor, 2004; Pomerantz et al., 2007). On the other hand, the parent-teacher relationships is a predictor of preschool children’s social behavior problems (Izzo et al., 1999; Epstein and Sheldon, 2002; Sheridan et al., 2012).

Building on the preceding analyses, this study proposes Hypothesis 1: Parent-teacher relationships is significantly associated with the preschool children’s social behavior problems.

### Teacher-parent relationships, parents’ work–family conflict and preschool children’s social behavior problems

A diverse array of environmental elements influence children’s development. Beyond factors within the microsystem and mesosystem, the characteristics of parents’ employment, a component of the exosystem, are intricately linked to the preschool children’s social competence (Tudge et al., 2017). The spillover-crossover model of emotion suggests that an individual’s emotions, attitudes, and behaviors in the work (or family) domain can spill over into the family (or work) domain. This process influences the emotions, attitudes, and behaviors of other members through social interactions (Ilies et al., 2017; Zhang and Lin, 2020). When balancing pressures from both work and family becomes challenging in certain aspects, individuals may experience an inter-role conflict known as work–family conflict. This typically manifests in two primary forms: work-interfering-with-family conflict and family-interfering-with-work conflict (Jung and Kim, 2023). For instance, characteristics of the workplace, including work hours, intensity, and the ratio of input to return, can affect an individual’s functioning at home. Research indicates that parents’ work–family conflict is a predictor of preschool children’s social behavior problems. Specifically, children of parents experiencing higher levels of work–family conflict are more prone to exhibit increased negative affect, encounter social adjustment challenges, and display hyperactive or inattentive behavior (Chai and Schieman, 2022).

A substantial majority (90–95%; Williams and Boushey, 2010) of preschool children’s parents experience stress or anxiety related to balancing work and family responsibilities. Work–family conflict is now a prevalent emotional concern among these parents. Kindergartens, as professional representatives of preschool education, have a broad functional scope. This includes enhancing the efficiency and scientific approach of parental childcare, as well as alleviating the emotional anxiety parents face regarding childcare. The notion of social support entails dual dimensions: the perceived availability of support and received support (Gleason and Iida, 2015). Perceived support is delineated as support readily available when required. Received support pertains to the tangible manifestation of a socially supportive interaction. This study predominantly delves into the dynamics of tangible social support interactions, with comparatively fewer analyses involving social networks or perceived support. Grounded in social support theory, a high-quality parent-teacher relationships can significantly help mitigate work–family conflict for parents of preschool children (Ryan et al., 2013). On a temporal level, aligning kindergarten entry and exit schedules, along with the timings for parents’ involvement in educational activities, with parents’ working hours can effectively reduce work–family conflicts (Ryan et al., 2013). From behavioral and emotional perspectives, parent-teacher cooperation firstly aids parents in mastering more scientific and effective educational strategies. This enhances the efficiency and scientific approach to family education, subsequently reducing work–family conflict (Gelfer, 1991). Secondly, parent-teacher exchanges facilitate a mutual comprehension of children’s behavior in varied settings by both teachers and parents. This understanding enables education based on a deep knowledge of the children, reducing parenting stress and anxiety, and thereby diminishing the level of work–family conflict experienced by parents (Gelfer, 1991).

It is evident that work–family conflict adversely impacts the preschool children’s social development. Conversely, a positive parent-teacher relationships contribute to reducing parents’ work–family conflict, thereby mediating its influence on preschool children’s social behavior problems. Based on the analyses, this study introduces Hypothesis 2: Work–family conflict serves as a mediator in the parent-teacher relationships and preschool children’s social behavior problems. In other words, the parent-teacher relationships is correlated with parents’ work–family conflict, which further correlates with the likelihood of preschool children’s social behavior problems.

### Teacher–parent relationships, parenting self-efficacy and preschool children’s social behavior problems

Parenting self-efficacy is defined as a parent’s perception of their ability to perform the expected behaviors associated with their parental role. In essence, it encapsulates a parent’s belief and assessment of their own parenting skills, representing the extent to which they feel confident about their parenting competence and behaviors (Liu et al., 2018). Parenting self-efficacy levels are shaped by social support factors, where higher levels of social support correlate with more effective parenting practices and enhanced parents’ health (Angley et al., 2015). For families, social support networks can be as crucial as the marital relationship (Guralnick et al., 2008). Parents typically experience reduced parenting stress and increased confidence in their parenting roles when they perceive adequate support (Angley et al., 2015). Research indicates that the quality of the parent–teacher relationships directly impacts parents’ motivational beliefs, encompassing various aspects of parenting self-efficacy and role construction (Kim et al., 2013). Notably, the level of perceived support positively relates to parenting self-efficacy (Shrooti et al., 2016), with the substantive support from teachers for parenting and parents’ perceptions of this support collectively influencing their parenting self-efficacy.

When parents embrace their role in educating their children as a personal responsibility and have confidence in their ability to fulfill this role, they are more inclined to engage actively in their children’s learning and development. This engagement can manifest in various forms, such as participating in family bonding activities at home or volunteering in kindergarten settings (Green et al., 2007). Research has revealed that elevated levels of parenting self-efficacy enhance a parent’s active involvement in their children’s health and education (Landeros, 2011). This heightened sense of responsibility leads parents to focus more on nurturing their children’s diverse skills and proactively preventing negative experiences in their children’s lives (Cinamon et al., 2007).

This highlights the correlation between parenting self-efficacy and children’s social development problems. As a significant determinant of parenting behavior, parenting self-efficacy is intimately connected to child developmental outcomes and their psychological adjustment (Jones and Prinz, 2005). Numerous studies have established that parents’ sense of parenting self-efficacy is linked to various facets of children’s developmental traits, encompassing their social adjustment (Albanese et al., 2019). Greater parenting self-efficacy is predictive of fewer behavior problems in children and is associated with enhanced social skills, better social adjustment, and overall improved child development (Albanese et al., 2019). Conversely, low parenting self-efficacy has been identified as a risk factor for negative parenting practices and children’s behavior problems (Albanese et al., 2019).

Drawing on these findings, this study posits Hypothesis 3: Parenting self-efficacy acts as a mediator in the parent-teacher relationships and preschool children’s social behavior problems. Specifically, the quality of parent-teacher relationships is associated with parenting self-efficacy, which further correlates with preschool children’s social behavior problems.

### Parents’ work–family conflict and parenting self-efficacy

Time and energy are crucial resources that significantly impact an individual’s ability to effectively meet their parental obligations (Adams and Golsch, 2023). While work–family conflict encompasses both work interfering with family life and family obligations disrupting work, it is more commonly observed that the demands of work have a greater impact on family life, leading to more frequent conflicts between these two important aspects of life (Eurofound, 2017). When parents encounter conflicts where family stresses seep into and disrupt their work life - a phenomenon known as family-to-work interference - they often blame themselves for ineffective parenting. This self-criticism can unfortunately lead to more strained and negative interactions with their children (Gali Cinamon et al., 2007). It is evident that heightened parental stress correlates with diminished parenting self-efficacy (Gordo et al., 2018). This stress adversely influences parenting behaviors, creating a negative feedback loop where poor parenting practices further escalate the stress levels of parents (Abidin et al., 2006). Work–family conflict represents a significant aspect of parental stress. Studies conducted in Australia, Hong Kong, and other parts of China have consistently shown a link between high levels of work–family conflict and reduced parenting self-efficacy and enthusiasm (Alexander and Baxter, 2005; Lau, 2010; Baxter and Smart, 2011). High level of work–family conflict predict low parenting self-efficacy (Gali Cinamon et al., 2007).

**Figure 1:**
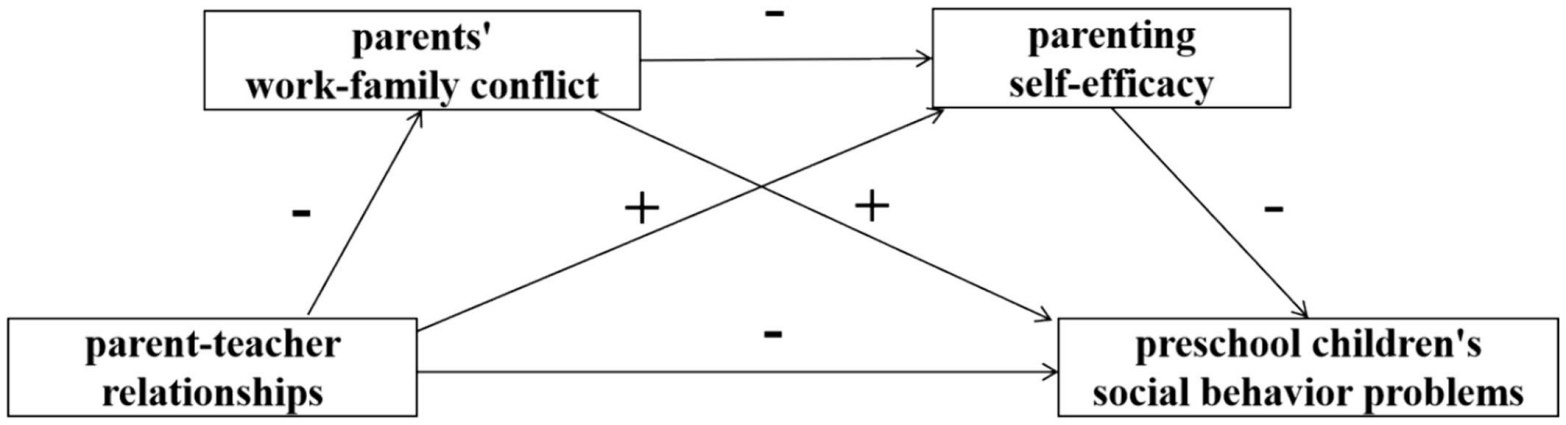
Hypothesized model of serial-multiple mediation of parents’ work–family conflict and parenting self-efficacy in the relationship between parent-teacher relationships and preschool children’s social behavior problems.

Drawing on ecological systems theory (Crawford, 2020), we recognize the complex interplay between the family environment and the broader social context. Parents’ work stress not only impacts their own well-being but also affects the family atmosphere and children’s social adaptability through emotional spill-over theory (Ilies et al., 2017; Zhang and Lin, 2020). Hence, work–family conflict is viewed as a significant environmental stressor that may indirectly influence children’s behavior and development by altering interactions and emotions within the family. Furthermore, parental self-efficacy represents parents’ assessment of their confidence and ability to fulfill their parenting roles (Liu et al., 2018). High-quality teacher-parent relationships can provide the necessary support and resources to enhance parental self-efficacy, thereby enabling parents to more effectively manage challenges at home and work, and mitigating adverse effects on children (Ryan et al., 2013). Such support and enhanced self-efficacy are crucial for fostering positive family educational practices and child development. In summary, this study posits Hypothesis 4: Parents’ work–family conflict and parenting self-efficacy serve as a sequential mediating link between the parent-teacher relationships and preschool children’s social behavior problems. It is observed that robust parent-teacher relationships, coupled with high parenting self-efficacy, can mitigate the onset of preschool children’s social behavior problems. Conversely, elevated levels of parents’ work–family conflict are predictive of the preschool children’s social behavior problems. Moreover, the parent–teacher relationships is theorized to impact preschool children’s social adjustment indirectly through a chain reaction initiated by parents’ work–family conflict and subsequently parenting self-efficacy. This hypothesis is encapsulated in a theoretical model that considers the layered and interactive elements within the child development ecosystem, as depicted in Figure 1. The choice of this theoretical framework aims to deepen our understanding of how family and school factors collectively influence child development, while providing empirical support for mitigating work–family conflict and enhancing parental self-efficacy to promote children’s social and behavioral health.

## Methods

### Participants

This study utilized a blend of purposive and convenience sampling methods to gather data from July 1 to July 25, 2023. Questionnaires were distributed to parents of children aged 3–6 years. The participants were sourced from four cities in Hebei Province, China - Qinhuangdao, Cangzhou, Handan, and Xingtai. To enhance the efficiency and quality of the questionnaire responses, the study engaged kindergarten principals or teachers to oversee the distribution process. The questionnaires were disseminated via an online platform. In an effort to minimize any external influences on the responses of participants, the questionnaire was designed to be anonymous. Additionally, all participants were informed about the objectives of the study and voluntarily consented to participate in the research. In this study, a total of 1,784 questionnaires were collected. Of these, 61 were discarded due to a consistent response pattern or a completion time of less than 3 min, resulting in 1,723 valid questionnaires. This amounts to a sample validity rate of 96.6%. Among the valid respondents, 1,404 were females (mothers, accounting for 81.5% of the sample) and 319 were males (fathers, making up 18.5%). The family structure of the respondents was as follows: 1,031 participants (59.8%) had an only child, while 692 (40.2%) had multiple children. In terms of educational attainment, 104 respondents (6.1%) had completed junior high school, 201 (11.7%) had finished senior high school, 528 (30.6%) possessed a college degree or higher, 781 (45.3%) held a bachelor’s degree, and 109 (6.3%) had obtained a master’s degree or higher. All research methodologies and procedures received the necessary approval from the review board of the institution affiliated with the first author (Table 1).

**Table 1:** Demographic characteristics of the subjects.

	Father (*N* = 319;18.5%)	Mother (*N* = 1,404;81.5%)	Total (*N* = 1723;100%)
Child gender			
Boy	159 (49.8%)	713(50.8%)	872 (50.6%)
Girl	160 (50.2%)	691 (49.2%)	851(49.4%)
Child age			
3 years	95 (29.8%)	432(30.8%)	527(30.6%)
4 years	98 (30.7%)	480 (34.2%)	578 (33.5%)
5 years	111(34.8%)	263 (28.5%)	473 (27.5%)
6 years	15 (4.7%)	130 (9.3%)	145 (8.4%)
Only child			
Yes	184 (57.7%)	847 (60.3%)	1,031 (59.8%)
No	135 (42.3%)	557 (39.7%)	692 (40.2%)
Single-parent family			
Yes	14 (4.4%)	21(1.5%)	35 (2.0%)
No	305(95.6%)	1,383(98.5%)	1,688 (98.0%)
Educational level			
Primary School and below	2 (0.6%)	8 (0.6%)	10 (0.6%)
Junior High School	14 (4.4%)	80 (5.7%)	94 (5.5%)
High School (including vocational)	30 (9.4%)	171 (12.2%)	201 (11.7%)
College	92 (28.8%)	436(31.1%)	528 (30.6%)
Bachelor’s Degree	158 (49.5%)	623 (44.4%)	781 (45.3%)
Graduate Degree and above	23 (7.2%)	86 (6.1%)	109 (6.3%)
Occupation			
Farmer	9 (2.8%)	16 (1.1%)	25 (1.5%)
Worker (including migrant worker)	47 (14.7%)	70 (5.0%)	117(6.8%)
Public Sector Employee (including civil servants and non-teacher public institution staff)	88 (27.6%)	299 (21.3%)	387 (22.5%)
Teacher and Research Personnel	13(4.1%)	145 (10.3%)	158(9.2%)
Corporate Manager	59 (18.5%)	126(9.0%)	185 (10.7%)
Military Personnel	1(0.3%)	1 (0.1%)	2 (0.1%)
Self-employed/Private Business Owner	53(16.6%)	315(22.4%)	368(21.4%)
Other	49 (15.4%)	432(30.8%)	481 (27.9%)

### Measures

To guarantee the reliability and validity of the research instrument, the scales employed in this study are reputable and well-established, sourced from prominent international journals. The instrument encompasses four distinct sections: the Parent-Teacher Relationships Scale, the Work–Family Conflict Scale, the Children’s Social Competence and Behavioral Assessment Scale, and the Parenting Efficacy Scale. These scales collectively provide a comprehensive measure of the variables under investigation.

### Parent–teacher relationships

The Parent-Teacher Relationships Scale (PTRS), developed by Vickers and Minke in 1995, was utilized for this study. This scale has two versions: one for parents and one for teachers. For the purpose of this study, the Parent-Teacher Relationships Scale (parent version) was employed. Comprising 24 questions, the scale evaluates the dimensions of “participation” and “communication.” Specifically, it explores six aspects of parents’ perceptions: a sense of belonging and support, reliability and availability, shared expectations concerning their children, communication tendencies, emotional sharing, and information exchange. The responses to scale questions are based on a 5-point Likert scale, ranging from 1 (almost never) to 5 (almost always). The questions include both positive statements (e.g., “I respect my child’s teacher”) and negative ones (e.g., “I do not like the way this teacher talks to me”).

### Parents’ work and family conflict

The Work and Family Conflict Scale (WAFCS), specifically developed for parents of children by Haslam et al. in 2014, was utilized in this study. This scale comprises 10 questions that assess two types of work–family conflict. The first type is work-family interference conflict, measured by 5 questions (e.g., “Work often makes me irritable or short-tempered at home.”). The second type is family-work interference conflict, also evaluated through 5 questions (e.g., “Household chores exhaust me and make it difficult to concentrate on work.”). Work–family conflict, arising from both work interfering with family and family interfering with work, can lead to the depletion of psychological resources, rendering it challenging for individuals to maintain optimal mental health. Extensive previous research has established that work–family conflict, which includes both types of interference, is inversely associated with mental health (Berkman et al., 2015; Yucel, 2017). Consequently, while this study primarily examines the effects of work–family conflict on the realm of family education, it concurrently assesses the intrusion of family on work. This is predicated on the understanding that by affecting parents’ mental health, such intrusions also indirectly influence their approach to family education. Responses to the scale questions are recorded on a 7-point Likert scale, ranging from 1 (completely disagree) to 7 (completely agree). It’s important to note that the scale does not include reverse scoring items. Therefore, a higher score directly indicates a higher level of work–family conflict experienced by the parent.

### Parenting self-efficacy

The Tool to Measure Parenting Self-Efficacy (TOPSE), developed and later refined by Kendall and Bloomfield in 2005, is a globally utilized scale for evaluating the effectiveness of various parenting programs by measuring parents’ perception of their parenting self-efficacy (Ferdowshi et al., 2021). This scale encompasses 48 questions, which are spread across 8 key dimensions: emotions and feelings, play and enjoyment, empathy and understanding, control, discipline and boundaries, stress, self-perception, learning, and knowledge. Each dimension is represented by 6 questions. The responses are gauged on an 11-point Likert scale, with a range from 0 (completely disagree) to 10 (completely agree). The scale includes both positive and negative statements. The total score, obtained by summing the scores of all questions, indicates the level of parenting self-efficacy; a higher total score signifies greater parenting self-efficacy.

### Social competence and behavior

The Social Competence and Behavior Evaluation Scale-30 (SCBE-30) for children, created by Lafreniere and Dumas in 1996, was employed in this study to assess preschool children’s social behavior problems. This scale has been validated and found to possess strong reliability in the Chinese context (Liu et al., 2012). Parents of the children reported on the scale, which comprises 30 items rated on a 6-point Likert scale ranging from 1 (never) to 6 (always). The SCBE-30 evaluates children’s social adjustment across three dimensions: sensitive cooperation (e.g., “My child is able to negotiate and resolve conflicts”), angry aggression (e.g., “My child is irritable and prone to temper tantrums”), and anxious withdrawal (e.g., “My child spends a lot of time alone and does not get along with others”) (Liang et al., 2022). In this study, the focus was specifically on preschool children’s social behavior problems. Consequently, two dimensions were emphasized: angry aggression and anxious withdrawal. Higher combined scores in these two dimensions indicate a greater frequency of social behavior problems in preschoolers.

### Control variables

The study incorporated several control variables, including parents’ gender, education level, occupation, the age of their children, and the number of children they have.

### Methods of analysis

Initially, descriptive statistical analyses of all variables were conducted using SPSS 26.0. This process involved determining the basic characteristics and distribution of the data by calculating means, standard deviations, and other relevant statistics for the main variables. Following this, the relationships among the four key variables—parent–teacher relationships, parents’ work–family conflict, parenting self-efficacy, and preschool children’s social behavior problems—were examined using Pearson’s correlation coefficient to assess their interconnections. Subsequently, model and path analyses were carried out using the SEM software Mplus. These analyses aimed to elucidate the direction and significance of the mediating effects, employing statistical results such as path coefficients, confidence intervals, and significance levels to interpret the findings.

## Results

### Reliability test and common method bias test

In this study, the reliability of the scales was assessed using Cronbach’s α coefficient and Composite Reliability (CR). As illustrated in Table 2, the Cronbach’s α values for the variables of parents’ work–family conflict, parenting self-efficacy, parent-teacher relationships, and preschool children’s social behavior problems were 0.927, 0.826, 0.929, and 0.868, respectively. Correspondingly, the CR values were 0.888, 0.808, 0.863, and 0.825. Each of these values exceeded the threshold of 0.8, indicating a high level of scale reliability. Furthermore, the Average Variance Extracted (AVE) for all variables was above 0.5, suggesting strong convergent validity. Discriminant validity in this study was evaluated using the Fornell and Larcker criterion, as detailed in Table 3. The results showed that the square root of the AVE value for each latent variable was higher than the correlation coefficients in the corresponding row, confirming that the model possesses robust discriminant validity.

**Table 2:** Results of construct reliability and validity tests for variables.

Variables	Cronbach’s alpha	CR	AVE
M1 parents’ work–family conflict	0.927	0.888	0.798
M2 parenting self-efficacy	0.826	0.808	0.767
X parent-teacher relationships	0.929	0.863	0.519
Y preschool children’s social behavior problems	0.868	0.825	0.702

**Table 3:** Results of discriminant validity tests for variables (Fornell and Larcker Criterion).

	M1 work–family conflict	M2 parenting self-efficacy	X parent-teacher relationships	Y social behavior problems
M1 work–family conflict	0.893			
M2 parenting self-efficacy	−0.199	0.814		
X parent-teacher relationships	−0.337	0.407	0.720	
Y preschool children’s social behavior problems	0.316	−0.210	−0.311	0.838

To mitigate common method biases in this study, where survey data were collected through self-reports from the children’s parents, procedural controls were implemented. These included conducting anonymous surveys and employing reverse scoring for some questions. Additionally, the Harman single-factor test was used to assess the presence of common method bias. The test results revealed 12 factors with eigenvalues greater than 1. Notably, the cumulative variance explained by the first factor was only 18.787%, falling well below the critical threshold of 40%. This indicates that the study did not suffer from severe common method bias (Zhou and Long, 2004). Furthermore, to evaluate the presence of multicollinearity, the Variance Inflation Factor (VIF) test was applied to each variable in the regression model. The highest VIF value recorded for any variable in the model was 2.344, significantly lower than the commonly used benchmark of 10. This result suggests that multicollinearity was not a serious concern in the study.

### Descriptive and correlation analyses

Descriptive statistical analyses were conducted for variables including the parent-teacher relationships, parents’ work–family conflict, parenting self-efficacy, and children’s social behavior problems. The means and standard deviations of each variable are presented in Table 4. Correlation analyses revealed significant relationships among these variables. Specifically, the parent-teacher relationships was found to be significantly negatively correlated with both parents’ work–family conflict and children’s social behavior problems (*r* = −0.293, *p* < 0.001; *r* = −0.271, *p* < 0.001), and significantly positively correlated with parents’ sense of parenting self-efficacy (*r* = 0.431, *p* < 0.001). Additionally, parenting self-efficacy showed a significant negative correlation with both work–family conflict and children’s social behavior problems (*r* = −0.197, *p* < 0.001; *r* = −0.246, *p* < 0.001) (refer to Table 4). These correlations provide initial support for the four hypotheses postulated in this study.

**Table 4:** Means, standard deviations, and correlation coefficients of each variable.

Variables	M	SD	1	2	3	4
X parent-teacher relationships	83.20	8.98	1			
M1 work–family conflict	29.90	12.76	−0.293^***^	1		
M2 parenting self-efficacy	219.23	30.35	0.431^***^	−0.197^***^	1	
Y preschool children’s social behavior problems	30.27	8.05	−0.271^***^	0.324^***^	−0.246^**^	1

*N* = 1723, ***p < 0.001, **p < 0.01.

### Chain mediation model analysis

To effectively manage measurement error, this study employed structural equation modeling (SEM) for testing chained mediation effects. Utilizing parent-teacher relationships as the independent variable, and parents’ work–family conflict and parenting self-efficacy as mediator variables, the study focused on children’s social behavior problems as the dependent variable. For the mediation effect analysis, the Mplus software was utilized, and the bias-corrected nonparametric percentile Bootstrap test was applied, involving 5,000 resamples, to conduct the mediation effect test and estimate confidence intervals. The results indicate that the model fit indices are good (*χ*2/df = 2.29, RMSEA = 0.03, SRMR = 0.01, CFI = 0.99, TLI = 0.99). Table 5 presents the SEM path coefficients, while the structural model fit index, represented by the r^2^ coefficients, indicates the explanatory power of the endogenous latent variables. Table 6 displays the SEM path coefficients. All these values meet the statistical requirements, indicating that the variables have a significant level of explanatory capacity.

**Table 5:** Regression analysis of variable relationships in the mediation model.

	Standardized			*R* ^2^	*F*
	*β*	SE	*t*		
parent-teacher relationships→parents’ work–family conflict	−0.417^***^	0.033	−12.737	0.086^***^	162.137
parent-teacher relationships→parenting self-efficacy	1.378^***^	0.077	17.997	0.191^***^	203.047
parents’ work–family conflict→parenting self-efficacy	−0.183^***^	0.054	−3.387		
parent-teacher relationships→preschool children’s social behavior problems	−0.123^***^	0.023	−5.419	0.154^***^	104.315
work–family conflict→children’s social behavior problems	0.162^***^	0.015	11.052		
parenting self-efficacy→children’s social behavior problems	−0.036^***^	0.006	−5.513		
parent-teacher relationships→preschool children’s social behavior problems (total effect)	−0.244^***^	0.021	−11.687	0.073^***^	136.589

^***^p < 0.001, ^**^p < 0.01.

**Table 6:** Test of the chained mediating effect of parents’ work–family conflict and parenting self-efficacy.

Effect	Path relationship	Effect size	Bootstrap 95% CI	Relative mediation effect
Direct effect	Parent–teacher relationships → preschool children’s social behavior problems	−0.035	[−0.055,-0.020]	44.87%
Path1	Parent–teacher relationships → parents’ work–family conflict → preschool children’s social behavior problems	−0.031	[−0.047,-0.019]	39.74%
Path2	Parents’ work–family conflict → parenting self-efficacy → preschool children’s social behavior problems	−0.011	[−0.021,−0.001]	14.10%
Path3	Parent–teacher relationships → parents’ work–family conflict → parenting self-efficacy → preschool children’s social behavior problems	-0.001	[−0.002,-0.000]	1.28%
Total effect		−0.078	[−0.125,-0.040]	–

**Figure 2:**
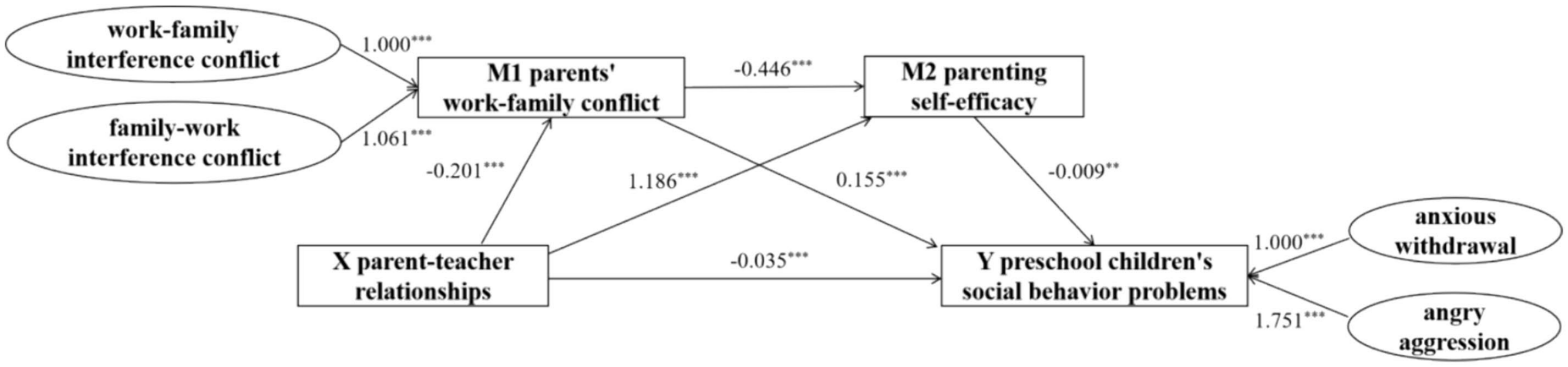
Chained mediation model of parent-teacher relationships affecting preschool children’s social behavior problems. ***p < 0.001, **p < 0.01. The β value represents the unstandardized raw regression coefficient.

Initially, the total effect test revealed that the parent-teacher relationships are significantly negatively correlated with preschool children’s social behavior problems, with an effect size of −0.043 (*p* < 0.001) and a bias-corrected confidence interval ranging from −0.057 to −0.030, excluding 0. This evidence supports the validity of Hypothesis 1. The model was subsequently expanded to include the mediating variables of parental work–family conflict and parenting self-efficacy, as illustrated in Figure 2. Analysis revealed that the indirect effect of parent-teacher relationships on children’s social behavior problems, mediated by parental work–family conflict, was −0.031 (*p* < 0.001), with a bias-corrected confidence interval from −0.047 to −0.019, also excluding 0 (path 1). This result supports the mediating effect of parental work–family conflict, corroborating Hypothesis 2. Additionally, the indirect effect of parent-teacher relationships on children’s social behavior problems, mediated by parental self-efficacy, was −0.011 (*p* < 0.001), with a bias-corrected confidence interval from −0.021 to −0.001, further excluding 0. This finding underscores the mediating role of parental self-efficacy, affirming Hypothesis 3. Moreover, the sequential mediation effect of parent-teacher relationships on preschool children’s social behavior problems through both parental work–family conflict and parenting self-efficacy was −0.001 (*p* < 0.001), with a bias-corrected confidence interval from −0.002 to 0.000, including 0. This result confirms the serial complete mediation effect of parental work–family conflict and parenting self-efficacy, thereby substantiating Hypothesis 4. These findings are elaborated in Table 6.

## Discussion

In the integrated framework of home-society co-education, the collective impact of families, kindergartens, and societal forces on the social development of preschool children is examined. Drawing from the ecological systems theory of child development (Crawford, 2020) and social support theory (Gleason and Iida, 2015), this study delves into the intrinsic connections between the family education subsystem of preschool children and the dynamics of parent-teacher and work-family relationships. It investigates the correlation between these relationships, particularly conflicts within them, and parenting self-efficacy, and consequently, the correlation with the development of social adaptability in preschool children. The study’s findings indicate that parents’ perceived positive parent-teacher relationships significantly enhance the social development of preschool children. It reveals that parents’ work–family conflict and parenting self-efficacy serve as mediating factors between parent-teacher relationships and preschool children’s social behavior problems. Although the indirect mediating effects are small, the chain-mediation effect of parents’ work–family conflict and parenting self-efficacy in the association between parent-teacher relationships and preschool children’s social behavior problems holds true, highlighting the multifaceted and interconnected nature of these relationships in the context of early childhood social development.

The association between parent–teacher relationships and preschool children’s social behavior problems.

This study found that there is a negative correlation between parent-teacher relationships and preschool children’s social behavior problems. Cooperation between kindergartens and families contributes to reducing children’s anger-aggression and anxiety-withdrawal. The findings confirm Hypothesis 1, aligning with the outcomes of earlier studies (Zhang et al., 2021; Wu, 2023). According to ecological systems Theory of child development, the parent-teacher relationships is an integral component of the child development ecosystem. With the ongoing economic transition and urbanization in China, there has been a gradual shift in public lifestyles. The dual extension of family and work hours, coupled with an increased demand for emotional investment, has exacerbated “time scarcity” and parenting anxiety among young urban parents. Consequently, there is a heightened need for comprehensive support from kindergartens, families, and communities. Epstein’s theory of school-family-community partnerships posits that establishing trust, communication, and satisfying deep-level interpersonal interactions are core to effectively forging partnerships among families, schools, and community members (Epstein, 2010). In the collaborative process of facilitating children’s learning and development, the quantity and frequency of interactions between parents and teachers lay the foundation for establishing strong parent-teacher relationships (Kim et al., 2013). However, the quality of the interactions is deemed more crucial than the frequency or quantity of contact (Patrikakou and Weissberg, 1998). The research findings of Adams & Christenson and Nzinga-Johnson & Baker support this perspective, suggesting that individuals perceiving positive parent-teacher relationships is a central driver for their effective involvement in children’s education. The quality of the parent-teacher relationship is identified as a key variable influencing parents’ active participation in their children’s educational processes (Adams and Christenson, 2000; Nzinga-Johnson et al., 2009). Therefore, in facilitating children’s social development, attention should be given not only to the external mechanisms such as the form and frequency of collaborative cooperation between families and kindergartens but also to the scientific nature and quality requirements of the cooperative education between families and kindergartens in terms of content and methods.

### Mediating role of parents’ work–family conflict

This study discovered that parent-teacher relationships influence preschool children’s social behavior problems through the impact on parents’ work–family conflict, aligning substantially with Hypothesis 2. This suggests that the work–family conflict faced by parents mediate the link between parent-teacher relationships and social behavior problems in preschool children. In the first stage of analyzing the mediating link, which focuses on the specific impact of parent-teacher relationships on parents’ work–family conflict, the study identified a negative correlation between them. This aligns with the outcomes of previous research (Ryan et al., 2013). For preschool children’s parents, support, guidance, and assistance in family education from kindergartens can help them balance their various life roles and reduce anxiety related to parenting (Barton et al., 2004). The degree of family-friendliness in institutions like kindergartens and work organizations plays a substantial role in facilitating individuals’ ability to balance work and family life (Voydanoff, 2005). Drawing on the emotional spill-over theory, this study further discovers that parents’ perception of parent-teacher relationships influences their emotional engagement and investment in family life and work tasks. When parents of preschool children receive support from kindergarten teachers in child-rearing, they tend to transfer the positive emotions and behaviors generated from parent-teacher interactions into their work and family systems with equivalent efficacy (Liu et al., 2021). This transference promotes a harmonious atmosphere within the family and facilitates the effective management of work-family relationships. The mutual support and co-operation between kindergartens, families, and work organizations contribute to the establishment of positive parent-teacher relationships and the balance between work and family life.

The second phase of the mediation process involves examining the predictive effect of work–family conflict on social behavior problems in preschool children. This study reveals a direct link between parents’ work–family conflict and social behavior problems in preschool children. It finds that parents’ work–family conflict is detrimental, impeding the healthy development of children’s social adaptability. For both fathers and mothers, elevated work–family conflict diminishes parents’ life satisfaction, escalates psychological distress, augments burnout risk, and intensifies depressive symptoms (McNall et al., 2009; Aarntzen et al., 2023). The heightened stress experienced by parents often permeates their parenting practices and emotional expressions, culminating in a tense home environment for young children. Within such an environment, children are prone to absorbing the stress around them, potentially manifesting in social difficulties like anxiety and social withdrawal. The persistent state of stress in parents, constituting a critical psychological micro-environment for young children, can be a direct precursor to behaviors like anger-aggression and anxiety-withdrawal in preschoolers’ social interactions. Consequently, parents who perceive a stronger parent-teacher relationship tend to experience lower work–family conflict. This, in turn, enables them to be more committed to fostering their children’s social competencies, leading to a reduction in social behavior problems among the children.

### Mediating role of parenting self-efficacy

This study reveals that the parent-teacher relationships as perceived by parents is significantly positively correlated with their sense of parenting self-efficacy. This finding aligns with the perspective of social support theory and is consistent with the research results of Kim et al. (2013). Parenting self-efficacy serves as a mediator linking a variety of factors, including parents’ characteristics, child traits, and situational elements (Junttila et al., 2007). Positive parent-teacher relationships and effective social support can reduce the stress associated with parenting, enhance parents’ involvement and confidence in parenting, and increase parenting self-efficacy. Consequently (Fang et al., 2021), these factors can influence parents’ family education practices and outcomes. Throughout the process where various microsystems and mesosystems influence the social development of preschool children, parents’ sense of parenting self-efficacy plays an important role as both a link and an agent of action.

Numerous studies have substantiated the correlation between parents’ sense of parenting self-efficacy and preschool children’s social adaptability and externalized behaviors (Cinamon et al., 2007; Angley et al., 2015). This study arrives at similar conclusions, indicating that parents’ sense of parenting self-efficacy is directly related to preschool children’s social development, with higher parenting self-efficacy predicting fewer social behavior problems. Parents with a strong sense of role construction and self-efficacy are more inclined to engage in educational activities such as assisting their children in learning various skills, communicating with teachers, and participating as parent volunteers (Green et al., 2007). In contrast, parents experiencing high parenting stress often perceive the demands of child-rearing as more extensive. They encounter heightened challenges while engaging in daily parenting activities to meet their children’s needs (Giallo et al., 2013). This elucidates the correlation between parenting self-efficacy in upbringing and preschool children’s social behavior problems.

### Chain mediating role of parents’ work–family conflict and parenting self-efficacy

The conclusion that parents’ perception of the parent-teacher relationships indirectly affects their sense of self-efficacy in parenting through work–family conflict is supported by empirical research findings. Belsky’s model of child-rearing processes posits that factors from three domains — parents, children, and social networks — collectively influence family education (Belsky, 1984). Although this study primarily examined the impact of parent-teacher relationships and work–family conflict on parental efficacy, it is crucial to acknowledge the proactive role of parents in navigating these relationships. Research indicates that effective parent-teacher communication can lead to parents developing a more scientific view of child development and education. This understanding often results in increased parental engagement in their children’s education. Consequently, this engagement fosters opportunities for positive relationships with teachers (Kohl et al., 2000). Parents may also be more likely to align their educational goals with those of the teachers (Christenson and Sheridan, 2001). Additionally, they might show a greater propensity for constructive and proactive communication and information sharing with teachers (Clarke et al., 2010). From this perspective, a heightened sense of parental efficacy also facilitates the establishment of positive parent-teacher relationships, further reinforcing the correlation between these relationships and parental efficacy in relation to child development.

Concurrently, it is essential to acknowledge that positive parent-teacher relationships not only significantly contribute to the advancement of family education for preschool children but also positively impact the effectiveness of preschool education (Syuraini et al., 2022). Strong parent-teacher relationships aid in providing a stable and supportive developmental environment for children, enhancing parental motivation and capability to engage in educational activities (Pirchio et al., 2023). This close co-operation between parents and teachers facilitates the sharing of information and the co-development of educational strategies tailored to the children’s developmental needs, thereby effectively supporting the enhancement of children’s social skills (Pirchio et al., 2023). Furthermore, higher quality preschool education further accelerates the progression of family education and child development, indicating that the influence of parent-teacher relationships on child growth is multifaceted and cumulative. Therefore, the significance of fostering positive co-operations between parents and teachers in promoting the comprehensive development of children is paramount, highlighting the importance of prioritizing the establishment of strong parent-teacher relationships.

## Limitations and implications

Although the collaborative educational approach involving family, community, and kindergarten has long been recognized and advocated, there is a notable scarcity of literature on the mechanisms through which parent-teacher relationships and work-family relationships, within the context of China’s economic and cultural backdrop, affect children’s development. There is a theoretical gap in studies considering parent-teacher relationships as predictive variables for the development of social skills in preschool children. Previous research on the antecedents of social development in preschool children has primarily focused on singular microsystems or subsystems, such as parenting styles or methods, teacher-child interactions, or teacher guidance. Empirical research exploring how the tripartite interaction among family, community, and kindergarten impacts family education and, subsequently, children’s development is exceedingly rare. Relative to the high emphasis and advocacy for collaborative work involving family, community, and kindergarten, the theoretical framework in this area has significantly lagged behind practical needs. This study discusses the dynamic effects of the children’s development ecosystem and the importance of family-community-kindergarten co-operation for the social development of preschool children. It transcends the previous research limitations that narrowly focused on examining the impact of one or two agents within the family-community-kindergarten nexus. By expanding from microsystems to outer systems, it clarifies the mechanism through which family-community-kindergarten co-operation influences family education. This provides reference for enhancing the quality of family education and promoting the social development of preschool children. The present study focuses on the potential factor changes within the ecosystem of preschool children’s development under the backdrop of family-community-kindergarten co-operation, and how these changes affect parenting self-efficacy, thereby influencing the social adaptation behaviors of preschool children. This holds significant referential value for the nascent empirical research on collaborative education involving family, community, and kindergarten.

The limitation of this study is that it did not encompass other intermediate and macro sub-systems beyond the parent–teacher relationship and work–family conflict. Future research could consider incorporating additional elements from intermediate and macro systems to further explore their relationships with various subsystems within the family system. Moreover, the study did not delve into the work characteristics of the target parents or the number of their children. Future research could address these aspects, including the number of children in a family and the associated parenting burdens. Although this study explored the correlation between adults’ Work–Family Conflict and Preschool Children’s Social Behavior Problems, it lacks analysis of the underlying causes of adults’ Work–Family Conflict. Future research should further investigate the relationship between adults’ job characteristics and Work–Family Conflict to better understand the correlation between adults’ job characteristics and children’s developmental characteristics. Additionally, this study employed a cross-sectional design to examine the connections between various levels of ecological subsystems within family-community-kindergarten co-operation and their association with the social behaviors of preschool children. Subsequent research could utilize a longitudinal design to investigate the directionality of these ecological subsystem relationships within family-community-kindergarten systems and whether these relationships evolve over time. At the same time, the study focused on the specific cultural context of China, without considering the characteristics of other cultural backgrounds. Therefore, the generalizability of the findings is limited. Future research conducted in cross-cultural contexts is of significant importance. The data for this study primarily relied on self-reports from children’s parents, which may introduce bias. This is also one of the limitations of the study and should be addressed in future research.

## Conclusion

Parent–teacher relationships and work–family conflict are associated through a “spill-over” effect, which is then related to parenting self-efficacy, indirectly being related to the preschool children’s social behavior problems. In the collaborative education process involving family, community, and kindergarten, parents’ cognitive and emotional experiences (parenting self-efficacy) serve as a bridge connecting macro, meso, and micro ecological systems with children’s behavior. An educational atmosphere in the family, grounded in harmonious parent-teacher and work-family relationships, stimulates parents’ positive emotions and behaviors, contributing to the improvement of the current state of family education.

Amidst the ongoing economic transformation and urbanization in China, there has been a gradual shift in the public’s lifestyle. Firstly, the rise in dual-income households is noteworthy. Young parents in these families are increasingly faced with the challenge of balancing work responsibilities and family duties to ensure the smooth functioning of their daily lives (Tong and Liu, 2015). Secondly, the increasing shift towards nuclear family structures often leaves young parents without the traditional support of children’s grandparents, compelling young parents to assume family responsibilities more independently (Ma, 2023). This shift poses distinct challenges for the parents of preschool children. When kindergarten support offers scientific advice and assistance in managing work-family relationships, thereby alleviating the parenting burden, it enhances parents’ confidence in their parenting abilities and consequently boosts their sense of parenting self-efficacy. Conversely, if the interaction with kindergarten teachers is limited to highlighting the children’s developmental deficiencies and family-related causes without providing scientific guidance, this can intensify parents’ feelings of guilt and anxiety about parenting, exacerbate work–family conflicts, and diminish their sense of parenting self-efficacy.

Article 39 of the Family Education Promotion Law of the People’s Republic of China mandates that “primary and secondary schools and kindergartens should incorporate family education guidance services into their work plans.” This clearly indicates that implementing high-quality family-kindergarten interaction and cooperation has become an essential component of the professional responsibilities of kindergarten teachers. The approach of enhancing family education quality through parent-teacher co-operation, thereby jointly promoting the learning and development of preschool children, is widely recognized and accepted (Hedges and Gibbs, 2005). From the perspective of parent-teacher relationships connotations, the interaction mechanism between families and kindergartens, along with parents’ perception of kindergarten support levels, collectively influence parenting self-efficacy. Hence, improving the quality of parent-teacher relationships should be approached from two aspects. On one hand, the rationalization of external forms such as interaction time, format, and themes is necessary. On the other hand, it requires establishing a positive communication tendency and shared expectations for the children, enabling parents to perceive the accessibility and reliability of kindergarten support. Through appropriate emotional and information sharing, parents’ sense of belonging and support can be enhanced. Concurrently, it is important to recognize that the parent–teacher relationships is bidirectional. The two-way connection between parents and teachers plays a crucial role in providing scientific guidance and fostering the learning and development of preschool children (Gelfer, 1991). A partnership that aligns with the best interests of the child can only be established by integrating the bidirectional links between “family-to-kindergarten” and “kindergarten-to-family” (Couchenour and Chrisman, 2013).

Further, policies that contribute to the wellbeing of children and families should be enacted. Firstly, work–family conflict presents a significant challenge for many families. Relevant policies should enhance support for flexible work arrangements, parental leave, and reliable childcare services to alleviate familial stress and enable parents to better balance work and home life. Secondly, educational policies should encourage and support the implementation of family education guidance services in early childhood education institutions, such as incorporating these services into their daily work plans and providing professional development opportunities for teachers and educators to learn effective family engagement strategies, thus promoting communication and cooperation mechanisms between families and schools. Thirdly, the establishment of a supportive community environment should be advocated, encouraging community resources and services to align with the needs of families and schools, thereby creating a robust support network centered on child and family welfare. This study believes that through these comprehensive measures, policymakers can create a more supportive, healthy, and beneficial growth environment for children and families.

## Data availability statement

The raw data supporting the conclusions of this article will be made available by the authors, without undue reservation.

## Ethics statement

The studies involving humans were approved by the ethics committee of Hebei Institute of International Business and Economics. The studies were conducted in accordance with the local legislation and institutional requirements. Written informed consent for participation in this study was provided by the participants' legal guardians/next of kin.

## Author contributions

GL: Conceptualization, Project administration, Writing – original draft. ZJ: Investigation, Resources, Supervision, Writing – original draft. XZ: Data curation, Investigation, Methodology, Software, Writing – review & editing. ZW: Formal analysis, Methodology, Software, Writing – review & editing. WL: Data curation, Resources, Visualization, Writing – review & editing.

## Footnotes

^1^“Preschool” stage includes ages from 3 to 6 years in China, it’s a time range of children’s kindergarten life from the beginning to the end. Preschool education is highly valued and gradually promoted by the government, but it is not part of compulsory education.

^2^“Family education” and “parenting” are sometimes used interchangeably in everyday contexts, but Family Education is more focused on the mental development and moral education of children, as well as preparing them for better living and learning in society; Parenting places more emphasis on the physical growth and emotional development of children, representing a more comprehensive care.
